# Effectiveness of undergraduate medical students training on LGBTQIA + people health: a systematic review and meta-analysis

**DOI:** 10.1186/s12909-024-05041-w

**Published:** 2024-01-16

**Authors:** Ana Macedo, Maria Aurindo, Cláudia Febra

**Affiliations:** 1 Faculty of Medicine and Biomedical Sciences (FMCB), University of Algarve, Edifício 2 – Ala Norte Campus de Gambelas, Faro, 8005-139 Portugal; 2 ABC Clinical Reseach Center, Algarve Biomedical Center (ABC), Edifício 2 – Ala Norte Campus de Gambelas, Faro, 8005-139 Portugal; 3 National Statistical institute of Portugal, Lisboa, Portugal; 4 Faculty of Medicine, University of Porto, Porto, Portugal

**Keywords:** LGBTQIA+, Meta-analysis, Medical education, Medical students, Sexual and gender minority (SGM)

## Abstract

**Background:**

Adequacy of learning models and their ability to engage students and match session’s objectives are critical factors in achieving the desired outcome. In this systematic review and meta-analysis, we assess the methodological approach, content, and effectiveness of training initiatives addressing medical students’ knowledge, attitudes, confidence and discrimination perception towards LGBTQIA + people.

**Method:**

PubMed, Web of Science, Medline and Scopus were searched to identify published studies, from 2013 to 2023, on effectiveness of training initiatives addressing medical students’ knowledge, attitudes, confidence and discrimination perception towards LGBTQIA + people. The risk of bias of the selected studies was assessed by the Medical Education Research Study Quality Instrument. Overall effect sizes were calculated using a Mantel–Haenszel method, fixed effect meta-analyses.

**Results:**

A total of 22 studies were included, representing 2,164 medical students. The interventions were highly diverse and included seminars, lectures, videos, real-case discussions, roleplay, and group discussions with people from the LGBTQIA + community. After the interventions, there was a significant improvement in self-confidence and comfort interacting with patients and in the understanding of the unique and specific health concerns experienced by LGBTQIA + patients.

**Conclusion:**

Our findings indicated that the outcomes of interventions training actions for medical students that promote knowledge and equity regarding LGBTQIA + people, regardless of their scope, methodology and duration, result in a considerable increase in students’ self-confidence and comfort interacting with LGBTQIA + patients, highlight the need for more actions and programs in this area promoting a more inclusive society and greater equity.

## Background

In recent years, several European and US-based organisations have released guidelines with the goal of reducing disparities and promoting health equity, particularly those involving the LGBTQIA + population (Lesbian, Gay, Bisexual, Transgender, Queer, Intersex, Asexual people. The plus sign represents people with diverse sexual orientation, gender identity, gender expression and sex characteristics who identify using other terms) [1]. Several studies showed that Sexual and Gender Minority (SGM) individuals have inferior health status, higher mortality and morbidity rates, and less access to health care [2–11]. This complex scenario derived from an intersectional framework penalizing LGBTQIA + individuals.

In a 2011 [12], the Joint Commission International outlined the necessity for healthcare organizations and their professionals to deliver high-quality out health care services while respecting the diversity of their patients in a patient-centred practice. To support trans-inclusive college health programs, the American College Health Association produced guidelines that provide detailed instructions on how to foster greater tolerance, inclusiveness, and more equitable health care, emphasizing the importance of developing strategies to close the gap [13, 14]. 

Several medical schools have attempted to take measures to reduce the gap, create a more equal health and empowerment SGM community, recognizing the fact that we live and are educated in a heteronormative society which is reflected in medical education. Integration of LGBTQIA + health training can significantly reduce health disparities and promote health equity [15–18].

The role of medical education in shaping the next generation of healthcare providers is critical. It is therefore essential that medical students receive comprehensive training that prepares them to provide effective medical care to all patients [19, 20].

In this context, there are many different interventions that are used in medical education, including traditional classroom instruction, hands-on clinical training, simulation-based learning, and the use of digital technology such as virtual reality [21–23]. Each of these interventions has unique strengths and weaknesses, and the choice of intervention will depend on the learning objectives, availability of resources, and the preferences of the students and faculty.

Since adult learning theories recognize that students have unique experiences, needs, and motivations that influence how they acquire new knowledge and skills, the use of lectures by itself is not sufficient to ensure that students comprehend and specially, retain information [24–26]. Therefore, other teaching methods that promote active learning and facilitate the development of critical thinking skills should be considered, including problem-based learning, team-based learning, and case-based learning [27–29]. 

Another critical issue is the inclusion of LGBTQIA + people in medical education centred in SGM health. The inclusion of individual histories and experiences in medical education is of paramount importance as it provides invaluable learning opportunities for healthcare professionals. Patients are the central focus of healthcare, and their experiences and perspectives can provide students with a unique insight into the challenges and complexities of healthcare delivery [30–33]. 

In this context, involving LGBTQIA + people in medical education initiatives helps to bridge the gap between theoretical knowledge and clinical practice, allowing that students can apply the knowledge and skills in real-life situations promoting a better understanding of the impact of their actions on patients’ health outcomes [34, 35]. It also promotes a more effective communication, empathy, and understanding of the patient’s preferences, values, and beliefs, and thus encourages a patient-centred approach in medical education. Patients can provide feedback on their experiences, which can be used to identify areas for improvement in healthcare delivery and curriculum design.

Finally, involving LGBTQIA + people in medical education can contribute to reducing stigma and discrimination, helping students to challenge their assumptions and biases and develop an open-minded and respectful approach to patient care [30, 36]. 

In this systematic review and meta-analysis, we assess the methodological approach, content, and effectiveness of training initiatives addressing medical students’ knowledge, attitudes, confidence and discrimination perception towards LGBTQIA + people.

## Methods

### Study design and inclusion criteria

The PRISMA guidelines (Preferred Reporting Items for Systematic Reviews and Meta-Analyses) [37] and the recommendations of the Cochrane Handbook were followed [38]. The included studies should meet the following criteria:

Population - medical undergraduate students.

Intervention - lecture, workshops, case studies, role play or discussion groups about SGM health.

Condition of interest - knowledge, attitudes, communication, discrimination and/or confidence regarding LGBTQIA + people health;

Outcomes - knowledge, attitudes, confidence, communication skill and/or discrimination outcomes measured by objective assessment instruments (including scales but also self-reported surveys, self-assessment of knowledge and attitudes).

### Literature sources and searches

The Web of Science, Medline, PubMed, and Scopus were searched for the following terms: (LGBT* OR gender minority OR sexual minority OR gay OR transgender OR gender identity) AND (medical OR medic*) AND (student* OR undergraduate* OR universit*) AND (train* OR action OR formation OR workshop OR class OR education) AND (knowledge OR communication OR discrimination OR confidence), limited to the last 10 years. The search was conducted on March 17th, 2023.

### Screening and data extraction

An initial screening of titles was performed by (AM) based on the inclusion criteria. Duplicates and studies that were clearly not related to the aims of this review were excluded. The abstracts were then screened independently by two reviewers (AM and CF) using the above criteria. The relevant studies and those for which the abstract raised doubts were independently assessed by the two reviewers in full text. All disagreements were resolved by consensus.

Information on study characteristics, design, intervention, participants, and outcomes was extracted from each of the studies. This data included authors, study date, university, sample size, medical year of students, study design, and interventions and outcomes. Zotero [39] was also used to obtain some publication data, such as titles, editors, URLs, digital object identifiers, page numbers, issue numbers, and volume numbers. The percentage of students who met the training program’s objectives was considered, together with the mean score change and standard deviations of the pre- and post-intervention.

### Risk of bias assessment

The quality of the studies was assessed by two reviewers, using the Medical Education Research Study Quality Instrument (MERSQI) [40, 41]. The MERSQI was developed to appraise methodological quality in medical education research and is suitable, reliable and simple to use. The comparison with the Newcastle-Ottawa Scale-Education (NOS-E) showed that both are useful, reliable, tools for appraising methodological quality of medical education researchco [40]. The MERSQI is composed of 10 items reflecting 6 domains of research quality (study design, sampling, type of data, validity, data analysis, outcomes).

### Meta-analysis

RevMan 5.4.1 (The Cochrane Collaboration) software was used. Likewise, although some studies assessed several different outcomes, in this meta-analysis, we included only the most frequent and homogeneous outcomes, namely students’ self-confidence and comfort interacting with LGBTQIA + patients and the understanding of the unique and specific concerns experienced in medicine by LGBTQIA + patients. The Mantel–Haenszel method was used. The standard mean difference (SMD) with 95% confidence intervals (CIs) was applied for the overall effect of group comparisons for continuous outcomes. The pooled odds ratio (OR) with 95% confidence interval (95% CI) was calculated to evaluate the percentage of students who met the intervention learning objectives. The statistical heterogeneity was calculated using the I^2^ statistic [42]. We set the significance level at 0.05 for pooled estimation results and built forest plots for each outcome.

## Results

**Fig. 1:**
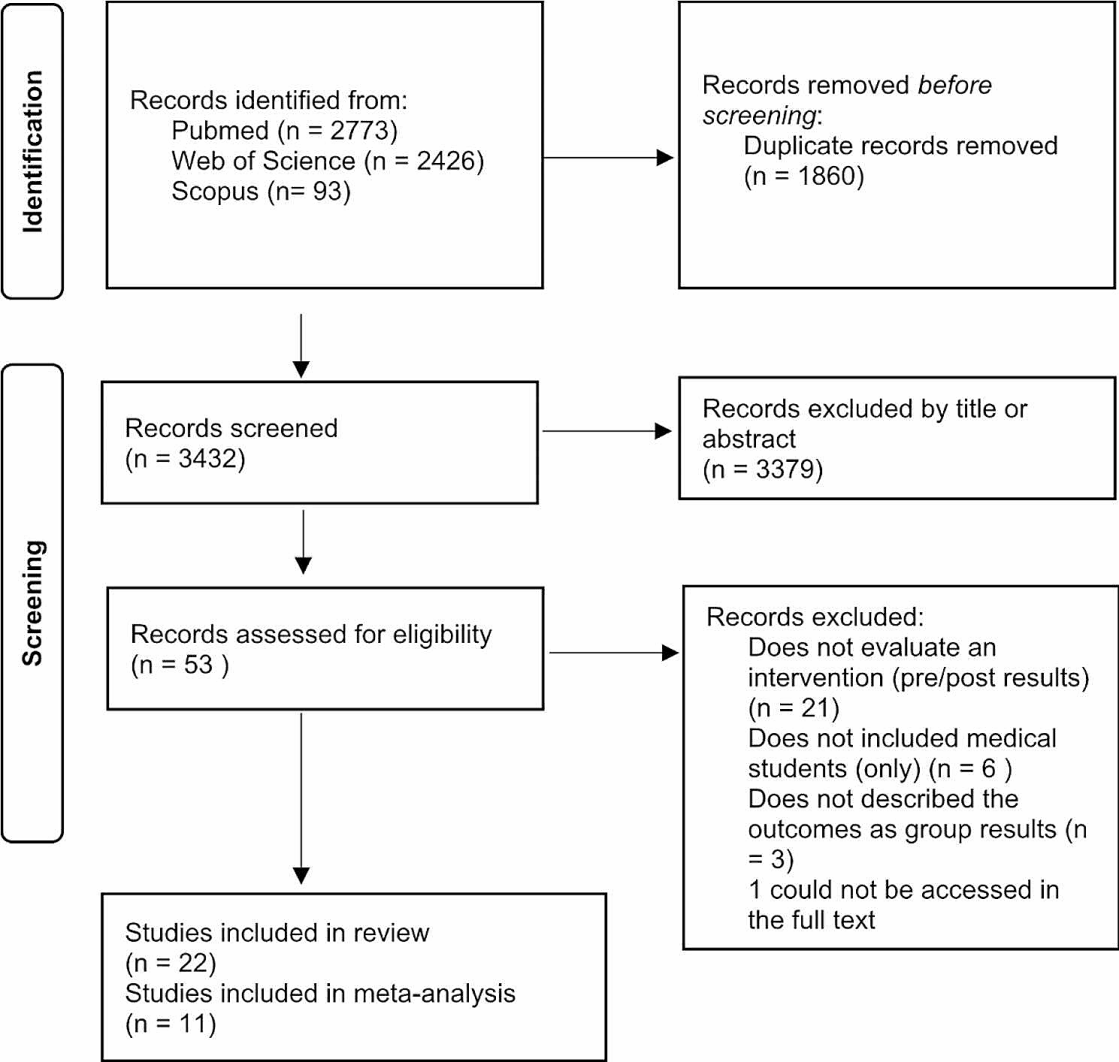
Study inclusion flowchart

From the 5,292 papers identified, 1,860 duplicates were removed manually, 3,379 articles were excluded based on title and abstract screening. Fifty-three full articles were then screened. However, 1 of them could not be accessed in the full text, and 30 articles were excluded against the inclusion criteria. Finally, 22 articles [43–64] were included for this systematic review (Figs. 1) and 11 were included in the meta-analysis [43, 44, 52, 53, 55, 59, 60, 60–63, 65].

### Synthesis of included articles

The studies evaluated 2,164 medical students, ranging from 1st to the final year, and were conducted in the United States, the United Kingdom, Ireland, Switzerland, the Republic of Korea, and Australia. The interventions were highly diverse, ranging from 1 to more than 10 h in several weeks and included seminars, lectures, videos, real-case discussions, roleplay, and group discussions with individuals from the LGBTQIA + community. Tables 1 and 2 summarize the studies’ characteristics, interventions, and results.

**Table 1:** Studies characteristics

Study	Focus	University/Country	Objectives	Intervention description
Arora, 2019^50^	TG	University of Newcastle (Australia)	To explore the effect of education on the confidence of students regarding TG care in Australia.	A multidisciplinary team, including a member of the TG community, delivered 3 times 1-hour sessions addressing appropriate TG terminology; exploring the biological basis of gender identity and diversity; the lived-experience of a TG and their relationship with healthcare providers; supportive care for children and families; adolescent puberty blockade; adult transition care; fertility; hormonal monitoring and surgery.
Berenson, 2020^51^	TG	Rutgers New Jersey Medical School (US)	To design a multimodal transgender curriculum to address educational gaps in the area of transgender health.	3 sessions: (1) a didactic presentation reviewing unique health issues and disparities experienced by TG focus on teaching students’ office-based masculinizing and femininizing therapies, (2) a small-group session viewing and analysing a pair of videos showcasing competent and poor communication between a provider and a TG, and (3) a large-group patient panel featuring members of the TG community.
Bi, 2020^52^	SGM	The University of Chicago Pritzker School of Medicine (US)	To evaluate the impact of an innovative module teaching intersectionality of sexual orientation, gender identity, and race/ethnicity issues	8-week course on Health Care Disparities: Equity and Advocacy. SGM health care disparities with a focus on intersectionality. 2-hour module: didactic presentation, role-play scenarios, and small-group work. 2.5-hour module with video interviews of SGM patients could complement modules that teach general SGM or race/ethnicity issues.
Click, 2019^53^	TG	East Tennessee State University (US)	To assess the effectiveness of the intervention on 1st - and 2nd -year medical students’ attitudes and knowledge of transgender health.	Sessions called Integrated Grand Rounds included cases co-presented to students by clinical and basic science faculty members. Brief didactic presentations are interspersed between live patient interviews and small group breakout sessions led by trained 3rd- and 4th-year student mentors. ‘Case of Transition’ was presented at Integrated Grand Rounds.
Cooper, 2018^54^	SGM	Baylor College of Medicine (US)	To evaluate an intervention on LGBT topics.	1-hour didactic lecture in a traditional classroom with approximately 180 students. The lecture was intended to be interactive. The facilitator for the session should be a content expert in LGBT health disparities, as well as familiar with the concept of social determinants of health.
Dale, 2022^55^	TG	Swansea University (UK)	To examine the impact of an education session on medical students’ comfort with their knowledge of and ability to address the health needs of transgender patients.	Three optional 20 min lectures, with time for questions. The presenters were a senior lecturer and researcher, a senior medical doctor, and a transgender activist. Topics included the spectrum of gender identity, using pronouns, and a broad overview of experiences of transgender people across the age spectrum and life course.
Gavzy, 2019^56^	SGM	Rutgers New Jersey Medical School (US)	To prepare trainees to address the needs of LGBT community members.	2.5-hours workshop: (1) Identify the 4 dimensions of human sexuality; (2) a sexuality survey to reflect on their own sexual identity, comfort in discussing sexual health, and homophobia/transphobia; (3) a didactic presentation to review health issues and disparities for LGBT individuals, (4) small-group sessions to analyse videos showcasing competent and poor communication between a provider and patient.
Lee, 2020^57^	TG	University of Ulsan College of Medicine, (Republic of Korea)	To evaluate attitudes toward TG among medical students and demonstrate that including lectures on transgenderism in curricula would have a positive impact on students’ attitudes.	The participants were given a lecture on “Understanding Gender and Transgenderism”. The lecture included discussions on the definition and core concepts of gender, gender dysphoria, transgenderism and related epidemiology and biology, psychiatric and social issues, health disparities, general primary care, hormone replacement therapy, and surgical options.
Lee, 2022^58^	TG	University College Dublin (Irland)	To assess medical students’ awareness of the health issues faced by transgender people and assess the impact of a 1-hour session on this topic on their awareness and comfort.	After positive expressions of interest, a didactic lecture was developed based on the latest literature. The lecture content was reviewed by the National Gender Service Ireland. Didactic slideshow presented to two separate groups of final year medical students. At the second lecture, a transgender man was invited to attend to provide a patient perspective on the topic.
Levy, 2021^59^	SGM	Nova Southeastern University (US)	To assess the impact of the activity on knowledge, confidence, and attitudes of medical students regarding LGBT healthcare.	2-hour active learning session. Small group, case-based discussions facilitated by members of the LGBT community, using clinical scenarios that enabled discussion around best practices for providing equitable healthcare to LGBT seniors: 10 minutes for introductions, 40 + 40 minutes for discussing case 1 and 2, 20 minutes for a large group debrief, pre-designed ‘prompt’ questions by the LGBT community facilitators.
Mahabamunuge, 2021^65^	SGM	New York Medical College (US)	To identify whether a student initiated lecture series on topics related to gender and sexual health leads to greater student comfort with discussing topics related to diverse sexual content.	Five ‘Gender and Sexuality in Medicine’ seminars. The lecture series included fourteen lectures presented by content area experts, including clinicians, patients, and community stakeholders. Lecture topics included intimate partner violence, STIs and stigma, puberty suppression in transgender children, contraception and family planning, female genital cutting, and mental health in SGM patients.
Minturn, 2021^61^	SGM	University of Colorado School of Medicine (US)	To evaluate the effectiveness of a 10-hour LGBTQ health curriculum at improving medical students’ self-confidence and knowledge in working with LGBTQ patients.	10-hour LGBTQ health-related curriculum. The course’s five 2-hour sessions took place in classrooms. The first session began with an introductory presentation covering LGBTQ-related terminology and techniques for taking an inclusive sexual history. An inclusive communication handout was provided. Students were divided into groups and performed role-played cases.
Najor, 2020^62^	SGM	Mayo Clinic Alix School of Medicine (MCASOM) (US)	To identify the learning needs of the students associated with gender expression and sexual orientation, assess the quality of the lecture, and attitude and knowledge at 1-year post lecture.	1-h lecture. Attendance was mandatory. The lecture included an explanation of the spectrum of identities associated with gender expression and sexual orientation, a broad overview of LGBT + health disparities, and the description of a patient scenario to demonstrate how subtle aggressions by medical staff may lead to less health care utilization and poorer treatment outcomes.
Norwood, 2022^63^	TG	University of Texas at Austin (US)	To evaluate the efficacy of a novel patient-centred, case-based educational intervention on TG healthcare competencies, TG healthcare training and knowledge of TG healthcare needs	45 min case-based educational intervention addressing the use of pronouns, recognise and correct ‘deadnaming’ in the medical chart, use appropriate language when taking a sexual history, address biases related to gender, sexual orientation, and sexual practices and conduct a patient history. The educational intervention started with a 5-minutes introduction of the six TG patient collaborators.
Pathoulas, 2021^64^	TG	University of Minnesota (US)	To investigate whether medical students’ comfort and familiarity with Gender-affirming hormone therapy could increase after a short interactive program.	1-hour didactic and interactive lecture on Gender-affirming hormone therapy: (a) the scope of practice required to provide Gender-affirming hormone therapy; (b) an informed consent model of care; and (c) the medical management of masculinizing and feminizing hormone therapy, including dosing of relevant medications. The interactive portion of the lecture consisted of role-playing.
Sanchez, 2022^44^	SGM	College of Medicine, University of Central Florida (US)	To assess the impact of LGBTQI+ -specific education on the attitudes of medical students regarding LGBTQI + communities.	2-hour human sexuality lecture with a focus on LGBTQI + health and healthcare. The content was delivered by the course director, an LGBTQI + researcher, a child psychiatrist, and a clinical psychologist focused on LGBTQI + care. Learner-to-facilitator ratio was approximately 30:1. Prior to class instruction, class lecturers shared their personal stories and their advocacy work with LGBTQI + communities (10 min).
Silverberg, 2021^45^	TG	Department of Medicine, Florida Atlantic University (US)	To introduce a standardized patient activity focusing on communication with transgender and gender nonconforming individuals.	For the first 20 min of the encounter, students interacted to obtain a complete history. The case focused on a patient who recently moved to the area and needed to establish care as well as to refill medications. The students then received direct feedback for the remaining 10 min. At the conclusion students reconvened for a large group panel discussion with all TG who participated in the session.
Stumbar, 2018^46^	SGM	The Florida Internat. University’s Herbert Wertheim College of Medicine (US)	To evaluate if an instructional format provided an effective way to teach medical students about the social determinants of sexual and reproductive health.	2-hour session was divided into two 1-hour blocks. After the conclusion of the lecture, three community members were invited to discuss their experiences with the health care system, particularly as these experiences related to their sex, sexual orientation, or gender identity. At our institution, this panel included a transgender woman, a middle-aged gay man, and an older heterosexual woman with HIV.
Taylor, 2018^47^	SGM	Faculty of Health Sciences, University of Bristol (UK)	To evaluate the effect of a half-day teaching session focused on LGBT health care.	1- hour lecture introducing legislation, health inequalities, and the health of the TG community. 90-minute workshop for groups of 15–20 students, focusing on consultation skills, homophobic or heterosexist language, and awareness of inequalities and stigma. Role-play where the peer facilitator acted as a patient with gender dysphoria attending a general practitioner. Students’ smaller groups to discussion.
Thompson, 2019^48^	TG	Rush University Medical Center (US)	To evaluate a gender-affirming healthcare curriculum for second-year medical students.	Online videos and lecture. 3-hour workshop included case-based learning centred on a gender nonconforming: (1) role-playing with different pronouns; (2) review of practice questions formatted to represent those seen on medical board examinations; and (3) discussion of case vignettes. On a separate day attended 2-hour panel discussions. Short video ‘Voices of Transgender Adolescents in Healthcare’.
Wahlen, 2020^49^	SGM	Lausanne University Hospital (Swisserland)	To assess the knowledge and attitudes of medical students regarding LGBT people; To evaluate the impact of a 1-hour lecture on adolescent LGBT health needs.	A compulsory one-hour lecture on sexual orientation and gender identity development during adolescence. The lecture focused on facts about health issues of LGBT adolescents and was given by a paediatrician experienced in adolescent health.
Zheng, 2022^43^	TG	Rutgers Robert Wood Johnson Medical School (US)	To evaluate a voluntary 2-hour modified jigsaw exercise on trans health care to optimize the structure for medical students.	A 2 h session was held in classrooms that allowed groups of five students discuss with one another transgender-specific questions covered gender versus sexual identity, common feminizing and masculinizing hormone regimens, and expected effects of hormone regimens.

LGBTQI+ - Lesbian, Gay, Bisexual, Transgender, Queer, Intersex; SGM –Sexual and gender minority people; TG – transgender people; UK – United Kingdom; US – United States of America

**Table 2:** Studies results and conclusions

Study	Evaluation methods	Students	Results	Original study authors’ conclusions	MERSQI (maximum 18 points)
Arora, 2019^50^	5-points Likert scale or as ‘true’ or ‘false’ to categorical statements. Pre/ Post session	3rd year medical students, *n* = 79	‘Agreed or strongly agreed’ - Pre/Post, *p* value - A FEMALE patient reports identifying as MALE since adolescence and requests hormonal therapy’ − 11%/ 33%, *p* = 0.001 ‘A 13-year-old patient who has entered puberty reports identifying with a sex different to that assigned at birth and requests help with transition’ − 14%/ 35%, *p* = 0.001; ‘A 55-year-old MALE to FEMALE patient who has been treated with hormones but has not undergone sex reassignment surgery should be offered prostate cancer screening’ − 68%/ 88%, *p* = 0.007; ‘I believe that hormonal and/or surgical therapies are appropriate for most transgender patients and should be provided to those patients who request them’- 49%/ 75%, *p* = 0.004.	Following the intervention, significantly more students felt confident to facilitate transgender health care for adults, adolescents, and children; and more students agreed that medical and surgical treatment should be offered to transgender patients if desired.	10.5
Berenson, 2020^51^	Satisfaction levels with the module. Self-Perceived Confidence; 5-point Likert scale. Pre/ Post session	2nd year medical students; *n* = 123	Pre/Post mean (1 to 5): Describe the unique health issues and disparities experienced by TG 1.6/ 2.8, *p* < 0.001; Describe medical transitioning and hormonal therapies for TG 1.1/ 2.5, *p* < 0.001; Describe best practices for promoting culturally competent and affirming care for TG 1.5 to 2.9, *p* < 0.001.	This multimodal approach using didactic sessions, video-based small-group case discussions, and patient panels were correlated with a significant increase in confidence regarding care for the transgender community.	10
Bi, 2020^52^	The 5-point Likert “1-not at all confident” to “5-completely confident”, self-assessed in knowledge of SGM patients’ barriers, intersectionality, and communication. Pre/ Post session	1st year medical students, *n* = 82	(#) Define the terms sex, gender, sexual orientation, gender identity, and expression 4.2(0.66)/ 4.5(0.55), *p* = 0.02; Define intersectionality 3.5(1.21)/4.4(0.61), *p* < 0.001; Define minority stress 3.5(0.98)/ 4.6(0.55), *p* < 0.001; Identify barriers to care for LGBTQ patients. 3.5 (0.91)/ 4.2 (0.60), *p* < 0.001. Ask LGBTQ patients about their identities. 2.9(1.09)/ 4.0(0.73), *p* < 0.001.	Our teaching module on intersectionality improved students’ knowledge of and confidence in caring for diverse patients.	10.5
Click, 2019^53^	9-item multiple choice and true or false knowledge questions. Comfort and attitude answer choices were based on a 5-point Likert-type scale, (1 = strongly disagree, 5 = strongly agree). Pre/ Post session	1st /2nd -year medical students, *n* = 138	(#) I am comfortable interacting with transgender people 3.81(0.92)/ 4.14(0.70), *p* < 0.01; I am comfortable with my knowledge base in providing care to the transgender population 2.4(0.83)/ 3.70(0.63), *p* < 0.01; I prefer not to treat transgender or gender non-conforming patients in my future practice 1.86(0.93)/ 1.73(0.92), *p* < 0.01	This study shows a significant effect of participating in a student-led half-day educational intervention on many facets of medical students’ attitudes and knowledge about transgender patients.	11
Cooper, 2018^54^	Ability to complete each of the lecture objectives, on a 10-point scale (1 = low, 10 = high). Pre/ Post session	3rd year medical students, *n* = 63	(#) Describe the unique health risks often encountered by LGBT and gender-diverse patients 5.8 (3.2)/ 8.1 (3.2), *p* < 0.01; Explain how stages of physical and identity development across the life span affect LGBT and gender-diverse patients 5.1 (4)/ 7.7 (3.2), *p* < 0.01; Describe factors that may underlie health care disparities experienced by LGBT and gender-diverse patients 5.0 (4)/ 7.9 (3.2), *p* < 0.01.	The didactic lecture was able to increase students’ knowledge of how social determinants impact the health of LGBT patients. The lecture can be incorporated into a longitudinal curriculum on LGBT health.	9.5
Dale, 2022^55^	A 6question survey selfassessed knowledge and comfort with transgender issues; 5point Likert scale, where 1 was strongly disagree and 5 was strongly agree. Pre/ Post session.	1st to final year medical students; *n* = 22	(#) I am comfortable with my understanding about TG 3.67(1.03)/ 4.39(1.20), *p* < 0.05; I am familiar with the issues faced in medicine by TG 2.44(1.25)/ 3.56(1.25), *p* < 0.05; I need more teaching on gender and TG issues 4.22(1.35)/ 3.89(1.23), *p* > 0.05; I have enough knowledge to feel comfortable seeing TG as a patient for a nongender related issue 3.17(1.50)/ 4.00(1.24), *p* > 0.05; I have enough knowledge to feel comfortable seeing TG as a patient for a gender related issue 2.17(1.47)/ 3.22 1.26), *p* < 0.05	Our study showed that an education session can increase medical students’ understanding of, and comfort at interacting professionally with transgender patients.	11
Gavzy, 2019^56^	Confidence in addressing each of the learning objectives. 1 to 4 points scale. Pre/ Post session	1st year medical students, *n* = 178	Evaluation of Self-Perceived Confidence in Define/compare terms 2.78/3.59, *p* < 0.001; Describe unique health issues/disparities 2.34/3.34, *p* < 0.001; Develop better practices 2.24/ 3.42, *p* < 0.001.	This workshop was effective in helping 1st-year medical students appreciate the spectrum of sexual diversity, health issues facing LGBT individuals, and better practices to promote affirming care.	10.5
Lee, 2020^57^	GTS 7-point Likert scale (1 strongly agree to 7 strongly disagree). Pre/ 4 weeks after the session	2nd -year medical students, *n* = 49	(#) GTS 92.35(24.52)/ 85.69 (23.73); Higher scores reflect more positive attitudes.	Although there was no significant attitude change after the lecture, those who had previous LGBT related education showed significantly positive attitudes at pre/postintervention surveys than those without.	10
Lee, 2022^58^	Survey assessing the impact of the teaching on the knowledge of and comfort in dealing with transgender health issues. Pre/ Post session	Final year medical students *n* = 57	Pre-lecture reported good understanding/ post-lecture - better understanding - what TG means − 80%/79%; Healthcare issues unique to TG − 10%/95%; Role of gynaecology in the care of TG patients – 18%/93%. Comfortable with history taking from a TG patient – 66%/Increased confidence − 91%.	Our results demonstrate that one-hour teaching session was effective at significantly improving students’ knowledge of and comfort with the healthcare needs of transgender people.	9.5
Levy, 2021^59^	17-item attitude, knowledge, confidence regarding senior LGBT individuals’ health status and healthcare. 5-point Likert scale: ‘strongly disagree’ to ‘strongly agree’. Pre/ Post session	1st -year medical students, *n* = 38	(#) I am confident in my knowledge about… ‘the barriers to health faced by LGBTQ + individuals’ 3.21(1.02)/ 4.05(0.66),*p* < 0.001); ‘the unique health issues for LGBTQ + individuals’ 3.16(1.00)/ 4.05(0.77),*p* < 0.001; ‘good practices for promoting competent care for LGBT individuals’ 3.34(1.02)/ 4.21(0.74), *p* < 0.001); ‘inappropriate practices that prevent competent care for LGBT individuals’ 3.21(1.04)/ 4.16(0.72),*p* < 0.001.	Our study data demonstrate the effectiveness of the small group, case-based discussion approach involving members of the LGBT community as facilitators to enhance the cultural competency of the medical students	12
Mahabamunuge, 2021^65^	Comfort discussing topics presented. 5-point rating scale as follows: “1 = very uncomfortable,” to “5 = very comfortable.” Pre/ Post session	All medical students 2018–20 (*n* = 152)	How comfortable are you talking to patients about issues related to sexuality? ‘Very confident’ - TG 29% [22, 36]/ 68%[59,77]; LGBT 49% [41, 57]/ 84%[77,91]; How comfortable are you discussing with patients? Medical Transition 22% [15, 28]/ 57% [48, 67].	Our findings demonstrate that student-initiated lecture series can improve medical student comfort discussing sensitive topics related to gender and sexual health.	8.5
Minturn, 2021^61^	4-point Likert scale on confidence and acquisition of knowledge related to LGBTQ health or true/false questions. Pre/ Post session	2nd year medical students, *n* = 42	(#) Sex anatomy and gender identity 2.41(1.1)/ 3.46(1.1), *p* < 0.01; Articulate health needs for LGB patients 2.22(1.2)/3.56(1.1),p 0 < 0.01; Articulate health needs for transgender patients 2.00(1.2)/3.50(1), *p* < 0.01; Culturally sensitive terminology 73%/ 90%, *p* = 0.036; Gender-affirming hormone therapy 51%/75%, *p* = 0.096; LGBT-related health risks 31%/ 43%, *p* = 0.999; Barriers to accessing care 40%(25-55%)/ 33%(18-47%), *p* = 0.999.	Our 10-hour LGBTQ health curriculum was effective at improving medical students’ self-confidence in working with LGBTQ patients but was less effective at increasing LGBTQ-related medical knowledge.	11
Najor, 2020^62^	A 21-question online survey, on the comfort level with treating TG patients and personal beliefs and experiences. Pre/ 1 week after the session (and 1 year after)	1st year medical students, *n* = 86	Students who were comfortable pre/post session: caring for TG 76%/ 91%, *p* = 0.0073; Aware that TG have unique health risks and health 99%/ 89%, *p* = 0.0043; Correctly identify a definition of gender 57%/ 67%, *p* = 0.19; Recognize the disproportionate burden of illness and socially determined barriers to health in TG 91%/ 96%, *p* = 0.21; Recognize that LGBT + status independently predicts less access to health care 97%/ 97%, *p* = 0.82.	1-hour lecture can increase the proportion of medical students who demonstrate positive attitudes and correct knowledge on TG patient care for at least a year.	10.5
Norwood, 2022^63^	5-items survey on the training on TG health and healthcare. 5point Likert scale, where 1 was strongly disagree and 5 was strongly agree. Two true/false questions. Pre/ Post session	2nd /3rd years medical students, *n* = 44	(#) Gender identity and using pronouns with gender-diverse patients − 2.92(0.84)/ 3.19(0.82), *p* = 0.048; Discussing sexual practices with gender-diverse patients − 2.78(0.76)/ 3.00(0.83), *p* = 0.103; 2.86 Define differences between sex and gender; gender expression and identity − 2.89 (0.75)/ 3.22 (0.83), *p* = 0.026; Identify and address communication patterns that adversely affect the care of gender-diverse patients − 2.69 (0.79)/ 3.17 (0.81), *p* < 0.001	Our data suggest that stand-alone educational interventions developed in collaboration with TG patient that include direct interaction improved soft skills and provide a needed forum for students to ask questions and dialogue.	11
Pathoulas, 2021^64^	Surveys addressing self-perceived preparedness and comfort with learning objectives using a 5-point Likert scale. Pre/ Post session	2nd -year medical students, *n* = 263	(#) I am familiar with how to use a dosing guide in gender-affirming hormone care 1.0(0.14)/ 3.5 (0.16), *p* < 0.001; I am familiar with different medication options in gender-affirming hormone care 1.5 (0.17)/ 3.8 (0.11), *p* > 0.001; I feel confident that I could find resources to provide gender-affirming hormone care 2.5 (0.19)/ 4.1 (0.11), *p* < 0.001; I am familiar with the idea of gender-affirming hormone care in a primary care setting. 2.9 (0.21)/ 4.3 (0.09), *p* < 0.001.	1-hour interactive lecture on GAHT increases medical students’ perceived familiarity and comfort with gender-affirming care in the primary care setting.	10.5
Sanchez, 2022^44^	CSUN - Attitudes Toward LGBTQ issues (20 questions). 5-point Likert scale (1 - strongly agree to 5 - strongly disagree). Pre/ 48 h after the lecture	1st -year medical students, *n* = 103	(#) Comfort with LGBTQI + Patient Interactions: Gay men 4.44(0.82)/ 4.35(0.96), *p* = 0.38; Lesbian women 4.34(0.88)/ 4.36(0.91), *p* = 0.84; Female-to-male TG 3.57(1.22)/ 3.73(1.15), *p* = 0.23; Male-to-female TG 3.56(1.18)/ 3.74(1.18),*p* = 0.17; One is born homosexual, straight, or bisexual 3.38 (1.13) / 3.49 (1.15), *p* = 0.32; Homosexual people cannot become heterosexual 3.66(1.04)/ 3.55(1.14), *p* = 0.24; One is born transgender 3.14(1.13)/3.39(1.07), *p* = 0.006; Gender and Sexuality − 3.32 (0.77)/ 3.43 (0.86), *p* = 0.05.	The findings support the incorporation of LGBTQI + instruction into medical curricula and suggest that educators may consider consulting pre-intervention data before teaching LGBTQI + health content.	10.5
Silverberg, 2021^45^	Likert scale survey addressing improved confidence in solicitation of a social history and negotiation of pronouns with transgender patients. Pre/ Post session	2nd year medical students *n* = 126	92.2% of students agreed that they felt more confident using their patient’s identified pronouns, with 67.4% asserting strong agreement. 95.4% of students agreed that that they felt more confident soliciting sexual history. In total, 95.4% of students indicated improved confidence with the overall experience of taking a history from a TG patient.	Students felt more confident using their patient’s identified pronouns and improved global confidence.	10.5
Stumbar, 2018^46^	Comfort with and beliefs about various aspects of sexual and reproductive health. Likert-type questions. A positive rank is any change on the scale that results in an increase in score. Pre/ Post session	1st /2nd -year medical students, *n* = 90	Ranked Students’ Responses- Positive/ Negative Mean Rank, p - I feel comfortable discussing a patient’s sexual history as it relates to issues of gender development and identity 25.0/ 21.4, *p* < 0.001; I feel comfortable treating people with a different sexual orientation than my own 14.6/12.6, *p* = 0.025; I feel comfortable treating people with a transgender identity 19.7/18.6, *p* < 0.001; LGBTQ + people face unique health concerns compared to heterosexual and cis-gender people 21.2/15.1, *p* = 0.129.	This instructional format provided an effective way to teach medical students about the social determinants of sexual and reproductive health. Students reported increased comfort and confidence related to the subject matter.	10.5
Taylor, 2018^47^	Short questionnaire scale 1–4 (1 being the lowest level of competency and 4 being the highest). Pre/ Post session	2nd year medical students, from 2012-15, *n* = 350	How prepared students felt to consult with LGBT patient − 69% of the students rated themselves at a competency level of 1 or 2 before the workshop, and after the workshop went on to rate themselves as a competency level of 3 or 4.	The sessions are useful for students in terms of improving awareness of health inequalities and enabling consultation skills practice in an informal environment.	6.5
Thompson, 2019^48^	A gender identity-adapted version of the SOPCS. 5-point Likert (1 = strongly agree, 5 = strongly disagree). Pre/ Post session	2nd year medical students, *n* = 129	(#) Total scale score 93.31(10.34)/ 103.31 (12.76), *p* < 0.001; Skills subscale − 18.87 (4.59)/ 27.38 (4.53), *p* < 0.001; Negative attitudes subscale − 14.54 (6.70)/ 15.38 (8.58), *p* > = 0.001; Knowledge subscale 28.98 (4.30)/ 31.31 (4.71), *p* < 0.001.	The curriculum improved students’ gender-affirming medical competency, knowledge, and skills. The development of a sustained, longitudinal curriculum is recommended in addition to the continuing education.	11
Wahlen, 2020^49^	Questionnaire − 28 statements; Likert scale ranging from 1 (strongly agree) to 5 (strongly disagree). ATHQ, SEKHQ, LGBT assessment scale, GTS. Pre/ One month following the course.	4th -year medical students; *n* = 117	(#) Attitudes 84.8 (13.6)/86.8 (15.4), *p* < 0.001; Knowledge 73.7 (18.1) / 87.9 (15.7), *p* < 0.001; Judgement 69.8 (16.5)/ 74.4 (18.8), *p* = 0.01; Experience 77.0 (16.5)/ 82.6 (16.8), *p* = 0.002	Our study suggests that even a 1-hour lecture can improve students’ knowledge about LGBT health needs.	10.5
Zheng, 2022^43^	Questionnaire - confidence discussing specific topics (0 to 100). Attitudes and beliefs 5- or 7-point Likert scale (1 = not at all comfortable or strongly disagree, and 5 and 7 = extremely comfortable or strongly agree). Pre/ Post session.	1st -year medical students; *n* = 33	(#) Knowledge 2.4 (1.6)/ 5.0 (1), *p* < 0.001; Gender identity 50.6(16)/ 70.9(16), *p* < 0.001; Hormone therapy 22.7(10)/ 58.1 (10), *p* < 0.001; Trans patients deserve the same level of care as cis patients 6.5(1)/ 6.8(0.7), *p* > 0.05; Comfortable being known among patients as clinician who treats trans patients 4.6 (0.7)/ 4.7 (0.7), *p* > 0.05	This 2-hour session encouraged students to actively discuss trans health care with one another. The cooperative learning was effective at disseminating knowledge and creating an enjoyable experience.	10

(#) Pre/ Post session mean (SD), *p* value

ATHQ - Attitudes Towards Homosexuals Questionnaire; CSUN - Customized version of the California State University Northridge; GTS - Genderism and Transphobia Scale; LGBTQI+ - Lesbian, Gay, Bisexual, Transgender, Queer, Intersex; SGM – Sexual and gender minority; SEKHQ - Sex Education and Knowledge about Homosexuality Questionnaire; SOPCS - Sexual Orientation Provider Competency Scale; TG – transgender people

Studies have shown that interventions, whether brief and simple, enhance students’ self-confidence in their knowledge, attitude, and capacity for interfacing with LGBTQIA + people.

The results concerning the level of knowledge are more heterogeneous. While some studies showed a significant increase in students’ knowledge regarding language comprehension, the use of suitable and inclusive language, and the ability to identify specific health conditions, other showed no significant changes in the level of knowledge after the interventions.

Both students and teachers valued the participation of LGBTQIA + community members in the discussion forums, roleplay scenarios, or as moderators.

### Quality: risk of bias in individual studies

Tables 2 and 3 displays the studies’ methodological quality scores. The mean consensus MERSQI score was 10.2 out of a maximum of 18. None of the articles attained a score of more than 12 points. However, 18 (78.3%) scored over 10 points, indicating good methodological quality, considering that our analysis includes only single centre nonrandomized studies.

**Table 3:** Medical Education Research Study Quality Instrument Results (*n* = 22)

MERSQI Domain	Response Item (Points)	Number of Studies (%)
Study design	Single group cross-sectional or single group post-test only (1)	0
	Single group pre- and post-test (1.5)	22 (100)
	Nonrandomized, 2 group (2)	0
	Randomized controlled trial (3)	0
Sampling: Institutions	1 institution (0.5)	22 (100)
	2 institutions (1)	0
	3 or more (1.5)	0
Sampling: Response rate	NA (—)	-
	< 50% or not reported (0.5)	4 (18)
	50–74% (1)	4 (18)
	> 75% (1.5)	14 (64)
Type of data	Assessment by study participant (1)	22 (100)
	Objective (3)	0 (0)
Validity evidence for instrument	NA (—)	-
Content	Not present (0)	1 (5)
	Present (1)	21 (95)
Internal structure	Not present (0)	3 (14)
	Present (1)	19 (86)
Relationship to other variables	Not present (0)	21 (95)
	Present (1)	1 (5)
Data analysis: Sophistication	Descriptive analysis (1)	22 (100)
	Beyond descriptive (2)	22 (100)
Data analysis: Appropriateness	Inappropriate (0)	1 (5)
	Appropriate (1)	21 (95)
Outcome	Satisfactions, attitudes, perceptions, opinions, general facts (1)	22 (100)
	Knowledge, skills (1.5)	9 (41)
	Behaviors (2)	0
	Patient/health care outcome (3)	0

MERSQI - Medical Education Research Study Quality Instrument Results; NA – Not applicable

### Meta-analysis

**Fig. 2:**
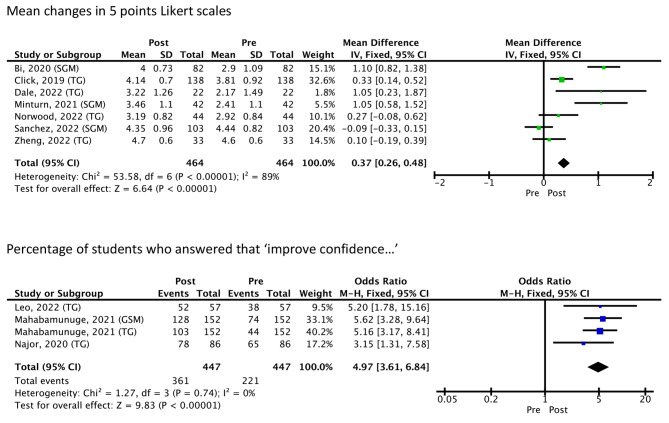
Students’ self-confidence and comfort interacting with LGBTQIA + patients. Mean changes in 5 points Likert scales. Notes: CI - Confidence interval; M-H - Mantel–Haenszel method; SGM – Sexual and gender minority people; SD - Standard deviation; TG - Transgender people

**Fig. 3:**
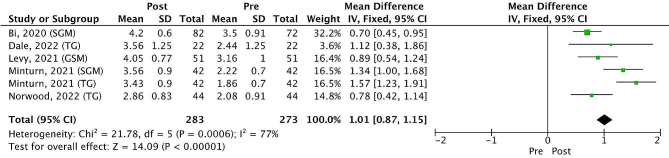
Understanding of the unique and specific concerns experienced in medicine by LGBTQIA + patients. Mean changes in 5 points Likert scales. Notes: CI - Confidence interval; M-H - Mantel–Haenszel method; SGM – Sexual and gender minority people; SD - Standard deviation; TG - Transgender people

The meta-analysis showed that after the interventions, there was a significant improvement in the students’ self-confidence and comfort interacting with LGBTQIA + patients. The mean difference across the 7 studies on a 5-point Likert scale pre-post test scores was 0.37 [0.26–0.48], p 0.00001, I^2^ = 89%. Four studies evaluated the percentage of students who answered that their confidence and comfort interacting with LGBTQIA + patients improved after the intervention. The results showed a significant improvement of 4.97 [3.61–6.84], *p* < 0.00001, I^2^ = 0% (Fig. 2). A significant improvement in understanding of the unique and specific concerns experienced in medicine by LGBTQIA + patients was found after evaluating five studies (six comparisons), with a mean difference on a 5-point Likert scale pre-post test scores of 1.01 [0.87–1.15], p 0.00001, I^2^ = 77% (Fig. 3).

## Discussion

In this review, we analyse the results of 22 studies, including 2164 medical students, regarding the effectiveness of interventions designed to promote knowledge and improve medical students’ confidence and comfort regarding level LGBTQIA + people. Overall, the studies had good methodological quality, with appropriate designs, scales, and statistical analyses, ensuring good internal validity. This is crucial to support the significance of our meta-analysis results.

The first aspect to be highlighted is the diversity of the interventions, both in terms of form, content, and duration. Overall, it was found that the level of knowledge about specific aspects of LGBTQIA + people health did not increase as expected after the interventions, but there was a significant increase in the students’ self-reported confidence and comfort. The analysis also shows that the interventions included moderators who were members of the LGBTQIA + community had very good results, emphasizing the importance of emotional and personal bonding beyond the simple transmission of theoretical content.

In recent years, the number of studies addressing the effectiveness of educational activities directed to medical students about LGBTQIA + people health specificities has significantly increased, translating the gap and the perceived need [30, 66]. 

This is probably the reflex of several statements and reports published by medical schools in the last decade. In 2014, the Association of American Medical Colleges published a document, entitled Implementing Curricular and Institutional Climate Changes to Improve Health Care for Individuals Who Are LGBT [67], which is based on the assumption that medical students have little or no information about SGM population in their formal academic curriculum, constituting a huge barrier to the future relationship with SGM people, and making urgent the need to incorporate this subject, in the curricula.

In our view, the incorporation of issues related to the health of LGBTQIA + people should be implemented by including LGBTQIA + people health specific themes immersivity in the medical curricula. The transversal incorporation of the theme, not focusing on one or the other disease, avoids the stigma associated to some pathologies, of which HIV infection is a paradigmatic example [68, 69]. 

In medical education concerning the health of LGBT people, it is crucial to emphasise that there are unique aspects, but also that many of the issues are transversal to all of society. In many conditions the impact is different, not so much by biological and clinical characteristics, but mainly by access to health care and social involvement, which determine asymmetries in health equity.

While the formal inclusion of the theme is critical, it does not obviate the implementation of more focused actions in specific themes, allowing the important direct contact with LGBTQIA + people and with life stories told in the first person, which is associated with a greater bond on the part of students, fundamental to increase their confidence and comfort in the clinical approach.

Several strategies can be utilized to better engage medical students in learning about LGBTQIA + people health and reducing health disparities in this community. First, it is vital to create a safe and inclusive learning environment that encourages open and respectful dialogue. This can be achieved by providing opportunities for students to share their own experiences and perspectives, as well as by inviting LGBTQIA + individuals to moderate the session and share experiences. Second, case studies, interactive simulations, and role-playing activities can be integrated to improve the understanding of the complex social and structural factors that affect LGBTQIA + people health. And, finally, providing ongoing support and mentorship to medical students can help ensure their continued engagement in addressing LGBTQIA + health disparities.

### Strengths and limitations

This systematic review includes more than 2000 students and gives an overview about the kind of interventions that have been done in the last decade regarding LGBTQIA + health in undergraduate medical education. One of the most important conclusions is that, above increasing the level of knowledge, the interventions carried out appear to improve students’ confidence and comfort in communicating with SGM people. This will improve the doctor-patient relationship, crucial for a true equity and inclusion.

In addition, our study has some limitations. First, the heterogeneity of interventions and outcomes evaluation methodology.

Second, the absence of control groups, which means that interventions can only be evaluated by a pre-post-test methodology, and as such biased by participants’ expectations. Third, the lack of long-term evaluation. Most studies only evaluate the outcome immediately after the intervention, giving no information on how long the outcome lasts over time.

## Conclusion

Our findings indicated that the outcomes of interventions training actions for medical students that promote knowledge and equity regarding LGBTQIA + people, regardless of their scope, methodology and duration, result in a considerable increase in students’ self-confidence and comfort interacting with LGBTQIA + patients, highlight the need for more actions and programs in this area promoting a more inclusive society and greater equity.

## Data Availability

The datasets used and/or analysed during the current study are available from the corresponding author on reasonable request.

## Abbreviations

CI: Confidence intervals (
LGBTQIA+: Lesbian, Gay, Bisexual, Transgender, Queer, Intersex, Asexual people. The plus sign represents people with diverse sexual orientation, gender identity, gender expression and sex characteristics who identify using other terms
MERSQI: Medical Education Research Study Quality Instrument
OR: Odds ratio
SGM: Sexual and Gender Minority
SMD: Standard mean difference
